# Laparoscopic versus open colectomy for locally advanced colon cancer in obese patients: a nationwide, multicenter, propensity score-based analysis of short- and long-term outcomes

**DOI:** 10.1093/jjco/hyae127

**Published:** 2024-09-22

**Authors:** Kentaro Nakajima, Tomonori Akagi, Yohei Kono, Hidefumi Shiroshita, Tetsuji Ohyama, Shuji Saito, Yoshinori Kagawa, Takatoshi Nakamura, Shinobu Ohnuma, Yutaka Kojima, Masafumi Inomata, Seiichiro Yamamoto, Takeshi Naitoh, Yoshiharu Sakai, Masahiko Watanabe, Japan Society of Laparoscopic Colorectal Surgery

**Affiliations:** Department of Surgery, NTT Medical Center Tokyo, Tokyo, Japan; Gastroenterological and Pediatric Surgery, Oita University Faculty of Medicine, Yufu, Oita, Japan; Gastroenterological and Pediatric Surgery, Oita University Faculty of Medicine, Yufu, Oita, Japan; Gastroenterological and Pediatric Surgery, Oita University Faculty of Medicine, Yufu, Oita, Japan; Biostatistics Center, Kurume University, Fukuoka, Japan; Division of Surgery, Gastrointestinal Center, Yokohama Shin-Midori General Hospital, Yokohama, Japan; Department of Gastroenterological Surgery, Osaka International Cancer Center, Osaka, Japan; Department of Gastroenterological Surgery, Osaka General Medical Center, Osaka, Japan; Department of Colorectal Surgery, Dokkyo Medical University, Shimotsuga, Japan; Department of Surgery, Tohoku University Graduate School of Medicine, Sendai, Japan; Department of Coloproctological Surgery, Juntendo University Faculty of Medicine, Tokyo, Japan; Gastroenterological and Pediatric Surgery, Oita University Faculty of Medicine, Yufu, Oita, Japan; Department of Gastroenterological Surgery, Tokai University School of Medicine, Kanagawa, Japan; Department of Lower Gastrointestinal Surgery, Kitasato University School of Medicine, Sagamihara, Japan; Department of Surgery, Osaka Red Cross Hospital, Osaka, Japan; Department of Surgery, Kitasato University Kitasato Institute Hospital, Tokyo, Japan

**Keywords:** colonic neoplasm, laparoscopy, body mass index

## Abstract

**Objective:**

This study evaluated the short-and long-term outcomes of laparoscopic colectomy versus open surgery in obese patients (body mass index ≥25 kg/m^2^) with locally advanced colon cancer to ascertain the non-inferiority of laparoscopic surgery to open surgery.

**Methods:**

In this large cohort study (UMIN-ID: UMIN000033529), we retrospectively reviewed prospectively collected data from consecutive patients who underwent laparoscopic or open surgery for pathological stage II–III colon cancer between 2009 and 2013. A comparative analysis was performed after propensity score matching between the laparoscopic and open surgery groups. The primary endpoint was the 3-year relapse-free survival (RFS).

**Results:**

We identified 1575 eligible patients from 46 institutions. Each group comprised 526 propensity score-matched patients. Comparing the laparoscopic versus open surgery group, laparoscopic surgery was significantly associated with increased median operating time (225 vs. 192.5 min; *P* < .0001) and decreased median estimated blood loss (20 vs. 140 ml; *P* < .0001). Lymph node retrieval (20 vs. 19; *P* = 0.4392) and postoperative complications (4.6% vs. 5.7%; *P* = 0.4851) were similar, postoperative hospital stay was shorter (10 vs. 12 days; *P* < .0001), and the 3-year RFS rates were similar (82.8 vs. 81.2%). The hazard ratio (HR) for relapse-free survival for laparoscopic versus open surgery was 0.927 (90% confidence interval [CI], 0.747–1.150, one-sided *P* for non-inferiority = .001), indicating that for obese patients with colon cancer, laparoscopic surgery was non-inferior to open surgery.

**Conclusion:**

Laparoscopic surgery in obese patients with colon cancer offers advantages in terms of short-term outcomes and no disadvantages in terms of long-term outcomes.

## Teaser

A retrospective large cohort study showed that laparoscopic surgery for obese colon cancer patients (body mass index, ≥25 kg/m^2^) offered advantages for short-term outcomes and no disadvantages for long-term outcomes.

## Introduction

Several reports including systematic reviews have compared the short- and long-term treatment outcomes of laparoscopic surgery for colon cancer in obese patients with a body mass index (BMI) of ≥30 kg/m^2^, or 25 kg/m^2^ versus non-obese patients (1–8). These reports found no significant differences between the obese and non-obese groups in short- and long-term outcomes. However, several studies have reported that laparoscopic surgery for colon cancer is technically more demanding in obese patients than in non-obese patients, and special care is required owing to the increased risk of postoperative complications (7,9). Additionally, in the JCOG0404 randomized controlled trial, which examined survival outcomes following laparoscopic versus open D3 lymph node dissection for stage II/III colon cancer, Saito et al. (10) showed that patients with a high BMI (>25 kg/m^2^) who underwent laparoscopic surgery tended to have worse survival rates than those who underwent open surgery, even after adjusting for clinicopathological factors. In this analysis, the type of recurrence could not be examined in detail owing to the insufficient number of cases, indicating the need for large-scale analyses with an adequate number of cases. Therefore, using the data of many patients undergoing surgery at facilities participating in the Japan Society of Laparoscopic Colorectal Surgery, we aimed to retrospectively analyze the short- and long-term outcomes and examine the technical and oncological safety of laparoscopic surgery in obese patients compared with patients who underwent open surgery using propensity score-matching analysis. We primarily aimed to examine whether laparoscopic surgery is non-inferior to open surgery in terms of relapse-free survival (RPS).

## Patients and methods

This study involved patients with pathological II/III colon cancer and a BMI ≥25 kg/m^2^ who underwent laparoscopic or open surgery at 46 institutions participating in the Japan Society of Laparoscopic Colorectal Surgery from January 2009 to December 2013. This cohort study and its associated protocols were registered in UMIN in 2018 (UMIN000033529). Although the study was retrospective, the expected 3-year RFS was set at the protocol planning stage, and the number of cases to be collected was calculated before the data were collected from multiple institutions. After obtaining approval from the institutional ethics committee, patient data were collected from each clinical report. The eligibility criteria were (i) colon cancer located in the cecum, ascending colon, transverse colon, descending colon, sigmoid colon, and rectosigmoid colon, (ii) pathological stage II/III with curative resection, 3) BMI ≥25 kg/m^2^, and (iii) age 20–79 years old. The exclusion criteria were as follows: (i) active multiple malignancies at diagnosis, (ii) cancer of the appendix, (iii) previous intestinal resection surgery (excluding appendectomy), or (iv) multiple colorectal cancer. Demographic and clinicopathological data included age, sex, BMI, comorbidities, pathological depth of tumor invasion, pathological lymph node metastasis, site of the primary lesion, estimated blood loss, operation time, degree of lymph node dissection, number of lymph nodes retrieved, number of lymph node metastases, postoperative complications, 30-day mortality, length of hospital stay, first recurrent organ, RFS, and overall survival.

The primary endpoint of this study was RFS. Secondary endpoints were overall survival, estimated blood loss, operative time, degree of lymph node dissection, lymph node retrieval, lymph node metastases, postoperative complications, 30-day mortality, length of hospital stay, and first-organ recurrence. Additional analysis verified long-term results for the open and laparoscopic groups according to location of the primary lesion, divided into the right-sided group (cecum, ascending colon, transverse colon) and left-sided group (descending colon, sigmoid colon, rectosigmoid colon), respectively, as well as treatment results for BMI divided into the BMI: 25–30 and BMI: ≥30 groups.

### Statistical analyses

The required sample size for the primary analysis was calculated to be 821 patients in each group, based on a non-inferiority margin of 1.373 for the hazard ratio (HR), a one-sided significance level of 0.05, and statistical power of 0.80. The non-inferiority margin settings preceded data collection. The power calculation was based on the outcome of JCOG0404. The 3-year RFS in JCOG0404 was 91.7% (95% confidence interval [CI], 85.2%–95.5%) in patients with BMI ≥25 kg/m^2^ (10). As the current study was a retrospective observational study, we expected the 3-year RFS to be lower than that reported in JCOG0404, which was 85% in the open surgery group and 80% in the laparoscopic surgery group, corresponding to a non-inferiority margin of 1.373 for HR. The appropriateness of allowing up to 5% inferiority was determined by clinical judgment after discussion among the investigators before data collection began.

Case matching was performed using the propensity score, which was estimated using a logistic regression model with the following 11 factors: age, sex, BMI, history of comorbidities (hypertension, diabetes mellitus, cerebrovascular disease, respiratory disease, and cardiovascular disease), depth of tumor invasion, lymph node metastasis, and sidedness, as prescribed in the protocol. Nearest-neighbor matching without replacement within a caliper was used. The size of the caliper was set to 0.2 of the standard deviation of the logit of the estimated propensity score. Patients who were found to be outside the caliper and unmatched patients were excluded. The degree of imbalance in patient characteristics was assessed using the absolute standardized difference (*d*), which is defined as $100\times \left|{\overline{x}}_1-{\overline{x}}_2\right|/\sqrt{\left({s}_1^2+{s}_2^2\right)/2}$, where ${\overline{x}}_1\ and\ {\overline{x}}_2$ are group means, and ${s}_1^2\ and\ {s}_2^2$ are group variances. We considered small (<10) values of d to support the assumption of balance between groups. All statistical analyses of the primary and secondary endpoints were performed for all matched-pair patients. The overall survival rate was calculated from the date of surgery until death from any cause or the date of the last follow-up. RFS was calculated from the date of surgery to that of confirmed recurrence or death from any cause. Survival curves were estimated using the Kaplan–Meier method, and the Cox regression model was used for group comparisons. Categorical variables were analyzed using Fisher’s exact test, and continuous variables were analyzed using the Wilcoxon test. A one-sided significance level of 0.05 was used. All *P* values in the secondary analyses were 2-sided, and *P* values <.05 were considered statistically significant. Propensity score matching and all other analyses were performed using R software version 4.02 (R Core Team, Vienna, Austria).

## Results

**Figure 1:**
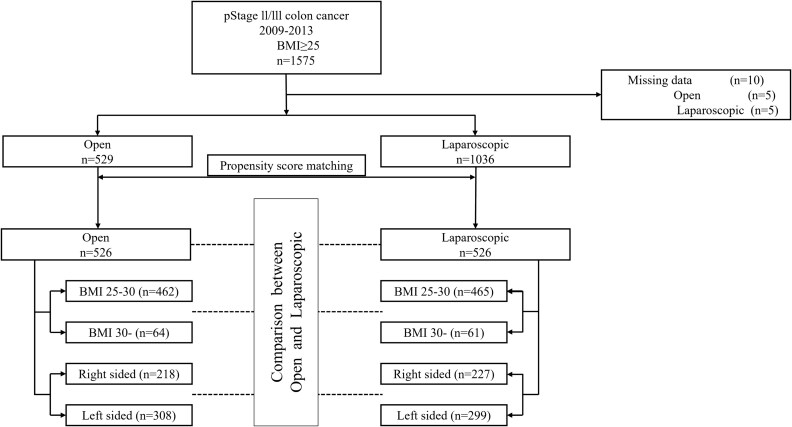
Flowchart of patient inclusion and exclusion and number of patients operated on. BMI, body mass index.

The data from 1575 patients were collected from 46 institutions. Among these 1575 patients, 1036 underwent laparoscopic surgery, and 529 underwent open surgery. After propensity score matching, 526 pairs of patients were included in the short- and long-term treatment outcome analyses (Fig. 1). The median follow-up period was 5.6 years.

The background data before matching showed that a history of cerebrovascular and respiratory diseases was often observed in the open surgery group, and the pathological depth of tumor invasion was deeper in the open surgery group (standardized difference was 23.87 before matching and 0 after matching). After matching, the mean BMI was similar between the two groups (laparoscopic vs. open, 27.5 vs 27.4 kg/m^2^) (Table 1). The primary lesion sites are listed in Supplementary Table 1.

**Table 1:** Patient and tumor characteristics

	Before PS matching			After PS matching		
	Open (n = 529)	LAP (n = 1036)	d	Open (n = 526)	LAP (n = 526)	d
Age, y, mean (SD)	65.3 (9.6)	65.2 (9.2)	0.53	65.2 (9.6)	65.3 (9.2)	0.40
Sex						
Female	223 (42.2%)	397 (38.3%)	7.82	222 (42.2%)	221 (42.0%)	0.38
Male	306 (57.8%)	639 (61.7%)		304 (57.8%)	305 (58.0%)	
BMI, kg/m^2^, mean (SD)	27.5 (2.4)	27.5 (2.6)	0.34	27.5 (2.4)	27.4 (2.5)	3.96
Comorbidity						
Hypertension						
No	271 (51.2%)	523 (50.5%)	1.49	270 (51.3%)	274 (52.1%)	1.52
Yes	258 (48.8%)	513 (49.5%)		256 (48.7%)	252 (47.9%)	
Diabetes mellitus						
No	398 (75.2%)	767 (74.0)	2.76	396 (75.3%)	386 (73.4%)	4.35
Yes	131 (24.8%)	269 (26.0%)		130 (24.7%)	140 (26.6%)	
Cerebrovascular disease						
No	486 (91.9%)	981 (94.7%)	11.27	486 (92.4%)	491 (93.3%)	3.69
Yes	43 (8.1%)	55 (5.3%)		40 (7.6%)	35 (6.7%)	
Respiratory disease						
No	483 (91.3%)	977 (94.3%)	11.62	483 (91.8%)	481 (91.4%)	1.37
Yes	46 (8.7%)	59 (5.7%)		43 (8.2%)	45 (8.6%)	
Cardiovascular diseases						
No	457 (86.4%)	910 (87.8%)	4.32	456 (86.7%)	459 (87.3%)	1.69
Yes	72 (13.6%)	126 (12.2%)		70 (13.3%)	67 (12.7%)	
Pathological depth						
Tis/T1/T2	27 (5.1%)	121 (11.7%)	23.87	27 (5.1%)	27 (5.1%)	0.00
T3/T4a/T4b	502 (94.9%)	915 (88.3%)		499 (94.9%)	499 (94.9%)	
Pathological lymph node metastasis						
N0	272 (51.4%)	536 (51.7%)	0.64	271 (51.5%)	271 (51.5%)	0.00
N1/N2/N3	257 (48.6%)	500 (48.3%)		255 (48.5%)	255 (48.5%)	
Sideness						
Right	219 (41.4%)	383 (37.0%)	9.08	218 (41.4%)	227 (43.2%)	3.46
Left	310 (58.6%)	653 (63.0%)		308 (58.6%)	299 (56.8%)	

PS, propensity score; d, absolute standardized difference; SD, standard deviation; BMI, body mass index.

Regarding intraoperative parameters, the operative time after matching was greater in the laparoscopic surgery group than in the open surgery group (median, 225 vs. 192.5 min, *P* < .0001) (Table 2A). The laparoscopic surgery group tended to have a lower estimated blood loss than the open surgery group (median, 20 vs. 140 ml; *P* < .0001). Based on the surgeon-reported degree of lymph node dissection, D3 lymph node dissection was performed significantly more frequently in the laparoscopic surgery group than in the open surgery group (88.2% vs. 77.6%, *P* < .0001). The median numbers of retrieved lymph nodes (laparoscopic vs. open, 20 vs. 19; =0.4392) and positive lymph nodes (0 vs. 0; *P* = .8208) showed no statistical difference between the two groups.

**Table 2A:** Operative outcomes.

	Before PS matching			After PS matching		
	Open (n = 529)	LAP (n = 1036)	*P* Value	Open (n = 526)	LAP (n = 526)	*P* Value
Estimated blood loss, ml			<.0001			<.0001
Median	140	20		140	20	
IQR	60.0, 300.0	10.0, 68.2		60.0, 300.0	10.0, 65.2	
Range	0.0–3205.0	0.0–2000.0		0.0–3205.0	0.0–1850.0	
Missing	0	2		0	2	
Operation time, min			<.0001			<.0001
Median	192	226.5		192.5	225	
IQR	156.0, 235.0	182.0, 278.0		156.0, 235.8	180.0, 275.0	
Range	60.0–537.0	80.0–1340.0		60.0–537.0	89.0–915.0	
Conversion to open surgery	NA	44 (4.2%)		NA	22 (4.2%)	NA
Degree of lymph node dissection			.0003			<.0001
D0	2 (0.4%)	2 (0.2%)		2 (0.4%)	1 (0.2%)	
D1	9 (1.7%)	5 (0.5%)		9 (1.7%)	1 (0.2%)	
D2	107 (20.2%)	144 (13.9%)		107 (20.3%)	60 (11.4%)	
D3	411 (77.7%)	884 (85.4%)		408 (77.6%)	463 (88.2%)	
Unknown	0	1		0	1	
Lymph nodes retrieved			0.7636			0.4392
Median	19	19		19	20	
IQR	13.0, 29.0	13.0, 27.2		13.0, 29.8	13.2, 28.0	
Range	2.0–120.0	1.0–79.0		2.0–120.0	1.0–79.0	
Lymph node metastases			.4737			.8208
Median	0	0		0	0	
IQR	0.0, 2.0	0.0, 2.0		0.0, 2.0	0.0, 2.0	
Range	0.0–44.0	0.0–32.0		0.0–44.0	0.0–32.0	
Surgery			<.0001			.0030
Right colectomy	161 (30.4%)	315 (30.4%)		161 (30.6%)	189 (35.9%)	
Transverse colectomy	39 (7.4%)	38 (3.7%)		38 (7.2%)	22 (4.2%)	
Left colectomy, Sigmoidectomy	223 (42.2%)	421 (40.6%)		221 (42.0%)	198 (37.6%)	
High anterior resection	58 (11.0%)	162 (15.6%)		58 (11.0%)	70 (13.3%)	
Low anterior resection	30 (5.7%)	84 (8.1%)		30 (5.7%)	41 (7.8%)	
Hartmann’s operation	8 (1.5%)	1 (0.1%)		8 (1.5%)	0 (0.0%)	
Others	10 (1.9%)	15 (1.4%)		10 (1.9%)	6 (1.1%)	

PS, propensity score; IQR, interquartile range. NA, not applicable

Among the short-term outcomes, postoperative morbidities (≥Clavien-Dindo grade 3) showed no statistical difference between groups (95.4% vs 94.3%, *P* = .4851) (Table 2B). Thirty-day mortality was not observed in either group. The median postoperative hospital stay was significantly shorter in the laparoscopic surgery group than in the open surgery group (10 vs. 12 days; *P* < .0001).

**Table 2B:** Short-term outcomes.

	Before PS matching			After PS matching		
	Open (n = 529)	LAP (n = 1036)	*P* Value	Open (n = 526)	LAP (n = 526)	*P* Value
Complication ≥C-D grade 3 (Overall)			.8214			.4851
No	499 (94.3%)	973 (93.9%)		496 (94.3%)	502 (95.4%)	
Yes	30 (5.7%)	63 (6.1%)		30 (5.7%)	24 (4.6%)	
Anastomotic leakage ≥C-D grade 3			.0052			.0903
No	525 (99.4%)	1010 (97.5%)		522 (99.4%)	516 (98.1%)	
Yes	3 (0.6%)	26 (2.5%)		3 (0.6%)	10 (1.9%)	
Unknown	1	0		1	0	
Ileus ≥C-D grade 3 (Overall)			.0472			.1159
No	518 (97.9%)	1028 (99.2%)		515 (97.9%)	522 (99.2%)	
Yes	11 (2.1%)	8 (0.8%)		11 (2.1%)	4 (0.8%)	
Obstructive ileus ≥C-D grade 3			.2354			1
No	525 (99.4%)	1033 (99.7%)		522 (99.2%)	523 (99.4%)	
Yes	4 (0.8%)	3 (0.3%)		4 (0.8%)	3 (0.6%)	
Paralytic ileus ≥C-D grade 3			.1203			.0693
No	522 (98.7%)	1031 (99.5%)		519 (98.7%)	525 (99.8%)	
Yes	7 (1.3%)	5 (0.5%)		7 (1.3%)	1 (0.2%)	
30-day mortality						
None	528 (100.0%)	1035 (100.0%)		525 (100.0%)	526 (100.0%)	
Missing	1	1		1	0	
Length of hospital stay, days			<.0001			<.0001
Median	12	10		12	10	
IQR	10.0, 16.0	8.0, 14.0		10.0, 16.0	8.0, 14.0	
Range	5.0–384.0	0.0–739.0		5.0–384.0	0.0–575	
Postop chemotherapy			.6440			.7140
No	260 (49.1%)	528 (51.0%)		259 (49.2%)	266 (50.6%)	
Yes	269 (50.9%)	507 (48.9%)		267 (50.8%)	260 (49.4%)	
Unknown	0 (0%)	1 (0.1%)		0 (0%)	0 (0%)	

PS, propensity score; C-D, Clavien-Dindo; IQR, interquartile range.

The 3-year RFS rate was similar between the two groups (laparoscopic vs. open surgery, 82.8% vs. 81.2%) (Fig. 2A). The HR for RFS for laparoscopic vs open surgery was 0.927 (90% CI, 0.747–1.150; one-sided *P* for non-inferiority = 0.001), indicating that for obese patients with colon cancer, laparoscopic surgery was non-inferior to open surgery. The post-matching 3-year estimated rate of overall survival for patients who underwent laparoscopic surgery was 95.0%, whereas that for open surgery was 94.1%. The HR for overall survival rate for laparoscopic versus open surgery was 0.976 (95% CI, 0.727–1.310) (Fig. 2B).

**Figure 2A:**
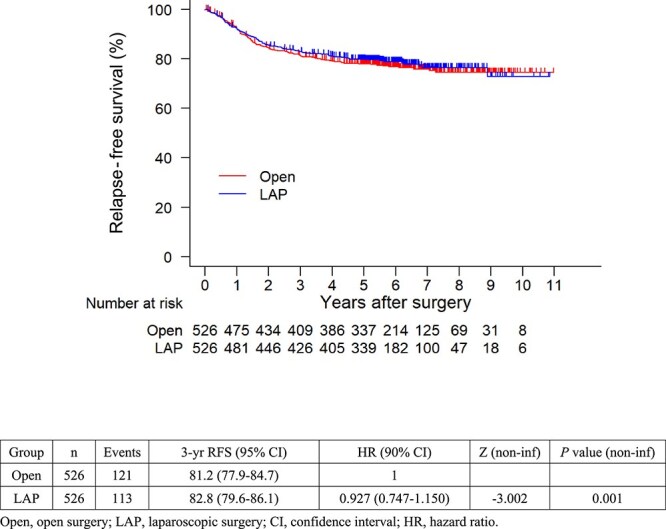
Kaplan–Meier plots for relapse-free survival (RFS) after propensity score matching. Open, open surgery; LAP, laparoscopic surgery; CI, confidence interval; HR, hazard ratio.

**Figure 2B:**
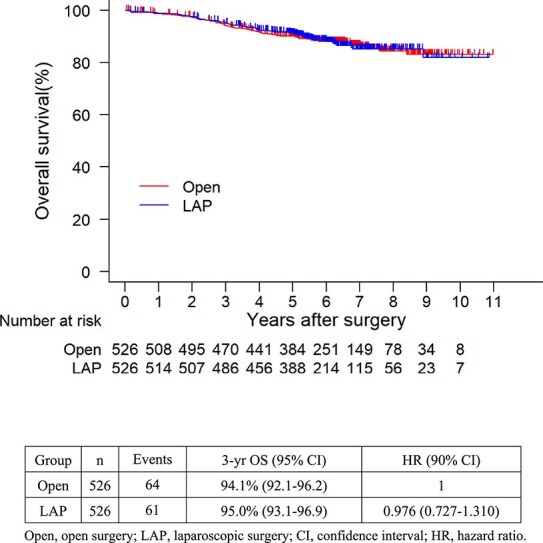
Kaplan–Meier plots for overall survival (OS) after propensity score matching. Open, open surgery; LAP, laparoscopic surgery; CI, confidence interval; HR, hazard ratio.

There were no differences in the sites of recurrence between the laparoscopic surgery and open surgery groups. No specific form of recurrence was found to be more common in either group (Supplementary Table 2).

Additional analysis of BMI subdivisions and tumor locations was performed. Among the laparoscopic surgery cases, more comorbidities such as hypertension, diabetes, cerebrovascular disease, and respiratory disease were found in the BMI: ≥30 group than in the BMI: 25–30 group. The conversion rate tended to be higher in the BMI: ≥30 group compared to the BMI: 25–30 group (13.1 vs. 3.0%, d = 37.8) (Supplementary Table 3). There were no significant differences in short-term outcomes between the two BMI groups in postoperative complications for either laparoscopic or open surgery (Supplementary Table 4). Long-term outcomes are shown for RFS (Supplementary Fig. 1A) and overall survival (Supplementary Fig. 1B) in the four groups: laparoscopic (BMI: 25–30, BMI: ≥30-) and open (BMI: 25–30, BMI: ≥30-). There was no significant difference in RFS between the four groups, but there was a trend towards poorer prognosis in the BMI: ≥30- group for open abdominal surgery.

The results of the additional analysis for tumor location showed no significant differences in RFS or overall survival between the four groups: open (cecum, ascending colon, transverse colon), open (descending colon, sigmoid colon, rectosigmoid colon), laparoscopic (cecum, ascending colon, transverse colon), and laparoscopic (descending colon, sigmoid colon, rectosigmoid colon) (log-rank test *P*-value for RFS = 0.781; overall survival = 0.711) (Supplementary Fig. 2AA and B).

## Discussion

Currently laparoscopic surgery remains an acceptable treatment option for colon cancer in Japan (9). However, the evidence for laparoscopic resection of colon cancer in obese patients is insufficient. The present study focused on locally advanced colon cancer of obese patients with a BMI ≥25 kg/m^2^ who underwent either laparoscopic or open surgery and is, to our knowledge, the largest study utilizing real-world data to date. In 2017, among patient subgroups with unfavorable long-term outcomes associated with laparoscopic surgery in the Japan Clinical Oncology Group Study JCOG0404, a randomized controlled trial comparing open and laparoscopic surgery for colon cancer, high BMI (>25 kg/m^2^) was reported to be associated with unfavorable long-term outcomes of laparoscopic colectomy (10). However, that study might not have provided concrete evidence of the superiority of open surgery over laparoscopic surgery for obese patients with colon cancer because of the small sample size and lower event rate. We thus conducted the present study to clarify the non-inferiority of laparoscopic surgery to open surgery among obese patients. BMI, which is easy to calculate, has been recommended as the measure to use to assess obesity for adults. A BMI of ≥30 kg/m^2^ is used to identify individuals with obesity by the World Health Organization (WHO) (11). However, the current WHO criteria to classify overweight and obesity in adult Europeans using BMI or waist circumference may not be appropriate in Asian populations. Indeed, it has been shown that the increased risks associated with obesity occur at lower BMIs in Asians and that these populations are predisposed to visceral or abdominal obesity. Therefore, WHO, the International Association for the Study of Obesity, and the International Obesity Task Force have proposed a lower BMI cut-off value for obesity in Asians of ≥25.0 kg/m^2^, which we used in the present study (12,13).

The results of the present large-scale propensity score-matching analysis showed laparoscopic surgery for obese patients (BMI ≥25 kg/m^2^) with colon cancer to be non-inferior to open surgery in terms of long-term outcome. In terms of short-term outcomes, intraoperative blood loss and length of postoperative hospital stay were better in the laparoscopic group. Despite the obese patients enrolled in the present study having numerous comorbidities, the surgeries were performed safely as a whole. The shorter length of postoperative stay in the laparoscopic surgery group of this study was consistent with the outcome from JCOG0404.

In the literature, the short-term results of laparoscopic surgery in obese patients with colorectal cancer have been reported as longer operative time (1,3,5–8), greater blood loss (1,8), higher conversion rates (3,7), more complications (1,6,7), and longer hospital stay (7) than in non-obese patients. However, other authors reported no significant differences with respect to blood loss (3–7), conversion rate (1,4,5,8), complications (3–5,8), and postoperative hospital stay (3,5). Some of these studies had BMI cut-offs of 30 (3,5,8) and 25 (6), whereas others compared three groups (BMI <25, 25 to 30, and ≥ 30 kg/m^2^) (1,7). Akiyoshi et al. concluded that laparoscopic surgery in obese patients with a BMI ≥30 kg/m^2^ was associated with significantly longer operating times, higher estimated blood loss, and increased postoperative complications compared with non-obese patients. Further, BMI ≥30 kg/m^2^ was independently predictive of anastomotic leakage. They also suggested that laparoscopic surgery for colorectal cancer in obese Japanese patients with a BMI ≥30 kg/m^2^ is feasible, but technically demanding and requires special care because of an increased risk of developing postoperative complications (1). Nakamura et al. stated that long-term oncological outcomes were similar in obese and nonobese patients (6). Two papers focusing on patients with higher BMIs provided a cautionary analysis. Park et al. (7) noted increased conversions to open abdominal surgery, longer operative times, and longer postoperative hospital stays in the very obese group (BMI >30 kg/m^2^) whereas Lee et al. (14) noted increased rates of in-hospital death, acute kidney injury, and deep vein thrombosis/pulmonary embolism, and a longer postoperative hospital stay in the more overweight group with a BMI ≥40 kg/m^2^.

In the sub-analysis of the present study, the short-term results showed no significant difference in postoperative complications between the two BMI groups for either laparoscopic or open surgery. Long-term results showed a trend toward poorer prognosis in the BMI >30 group for laparotomy when comparing the four groups (*P* = .711).

Regarding the choice between laparoscopic and open surgery, open surgery may have been chosen in more difficult cases for the three following reasons:

(1) Historical background: As the indications for laparoscopic surgery in obese patients are thought to have expanded in stages, it is possible that laparotomy was previously chosen for more difficult cases.
(2) Operator/institutional context: Surgeons familiar with and skilled in laparoscopy may have opted for this procedure, whereas surgeons who are still learning may have performed open laparotomy in difficult cases. Differences in patient backgrounds and outcomes between institutions should be investigated in future studies.
(3) Patient factors: Open surgery may have been chosen for patients with T4 perforation, obstruction, bleeding, or those requiring emergency surgery. However, these factors cannot be balanced by propensity score matching. Moreover, the presence or absence of comorbidities was considered as an adjustment factor for propensity score matching, but the severity of these comorbidities was not considered. For example, open surgery may have been performed in cases of severe cardiac and pulmonary decompensation.

The proportion of D3 lymph node dissections was significantly higher in the laparoscopic group than in the open group (86.2% vs. 77.9%; *P* = .0003). The degree of lymph node dissection is based on preoperative and intraoperative staging and is ultimately determined by considering the patient’s age, body type, and comorbidities. As this was a retrospective study, it is possible that there were differences between the institutions in the definitions of D2 and D3 dissections. The regions of lymph node dissection were defined by the Japanese Society for Cancer of the Colon and Rectum. According to Japanese guidelines, D3 dissection involves the removal of pericolic, intermediate, and main lymph nodes, whereas D2 dissection entails the removal of complete pericolic and intermediate lymph nodes (15). Further prospective studies verifying the photo documentation of dissection sites are needed to clarify the reason for the discrepancy in lymph node dissection between the two groups.

Our study has several strengths, including the number of patients analysed, large number of participating institutions, meticulous selection of only patients with a higher BMI and advanced colon cancer requiring D3 lymph node dissection, and the validated populations matched by propensity score, which we performed to balance the background patient data. Eleven factors were used, as described in the protocol and methods section. Matching resulted in ideally balanced patient background factors between the groups, making the comparison between open and laparoscopic surgery reliable. Moreover, the period between 2009 and 2013, during which the patients underwent colon cancer surgery, was almost aligned with current clinical practice compared to the period between 2004 and 2014 in JCOG0404. Because JCOG0404 cases were enrolled between 2004 and 2009, the enrollment period of this study (2009–2013) was designated as POST JCOG0404. Therefore, the results presented in this study can be interpreted as an improvement in surgical techniques in the JCOG0404 era.

Although there are some limitations, such as potential selection bias due to the retrospective cohort design of our study, this was minimized as much as possible using propensity score matching. As indicated above, the quality of complete mesocolic excision was not assessed in this study. However, as conceivable bias was reduced as much as possible, we believe that this study provides the highest level of evidence currently available on the validity of laparoscopic surgery in obese patients with locally advanced colon cancer.

## Conclusions

Laparoscopic surgeries for obese patients with stage II/III colon cancer were safely performed at 46 participating institutions. The incidence of postoperative complications was similar between the open and laparoscopic surgery groups, while the length of hospital stay was significantly shorter in the laparoscopic surgery group. The 3-year RFS rates in both groups were similar. Based on the results of this large cohort study, laparoscopic surgery may be considered a useful option, even for obese patients with advanced colon cancer.

## Supplementary Material

Supplementary_file_R3_3_hyae127
