# Supplementary material for: Laparoscopic versus open colectomy for locally advanced colon cancer in obese patients: a nationwide, multicenter, propensity score-based analysis of short- and long-term outcomes
Source: Jpn J Clin Oncol. 2024 Sep 22;55(1):21–8. doi: 10.1093/jjco/hyae127 (PMC11708209; doi:10.1093/jjco/hyae127)
Supplement: Supplementary_file_R3_3_hyae127 [file supplementary_file_r3_3_hyae127.docx]

| \| Supplementary Table 1. Site of the primary lesion \| \| \| \| \| \| \| \| --- \| --- \| --- \| --- \| --- \| --- \| --- \| \|  \| \| \|  \|  \|  \|  \| \|  \|  \|  \|  \|  \|  \|  \| \|  \| Before PS matching \| \| \| After PS matching \| \| \| \|  \| Open \| LAP \| *P* value^1^ \| Open \| LAP \| *P* value^1^ \| \| n=529 \| n=1036 \| n=526 \| n=526 \| \| Site of primary lesion \| \|  \| 0.001 \|  \|  \| 0.018 \| \| C \| 33 (6.0%) \| 90 (8.7%) \|  \| 32 (6.1%) \| 53 (10.1%) \|  \| \| A \| 106 (19.9%) \| 200 (19.3%) \|  \| 106 (20.2%) \| 120 (22.8%) \|  \| \| T \| 81 (15.3%) \| 93 (9.0%) \|  \| 80 (15.2%) \| 54 (10.3%) \|  \| \| D \| 36 (6.8%) \| 57 (5.5%) \|  \| 36 (6.8%) \| 28 (5.3%) \|  \| \| S \| 197 (37.2%) \| 391 (37.7%) \|  \| 195 (37.1%) \| 179 (34.0%) \|  \| \| RS \| 77 (14.6%) \| 205 (19.8%) \|  \| 77 (14.6%) \| 92 (17.5%) \|  \| \|  \|  \|  \|  \|  \|  \|  \| \| PS, propensity score; C, cecum; A, ascending colon; T, transverse colon; D, descending colon; S, sigmoid colon; RS, rectosigmoid colon.  ^1^Fisher’s Exact Test for Count Data with simulated *P* value (based on 2000 replicates) \| \| \| \| \| \| \| |
| --- | --- | --- | --- | --- | --- | --- | --- | --- | --- | --- | --- | --- | --- | --- | --- | --- | --- | --- | --- | --- | --- | --- | --- | --- | --- | --- | --- | --- | --- | --- | --- | --- | --- | --- | --- | --- | --- | --- | --- | --- | --- | --- | --- | --- | --- | --- | --- | --- | --- | --- | --- | --- | --- | --- | --- | --- | --- | --- | --- | --- | --- | --- | --- | --- | --- | --- | --- | --- | --- | --- | --- | --- | --- | --- | --- | --- | --- | --- | --- | --- | --- | --- | --- | --- | --- | --- | --- | --- | --- | --- | --- | --- | --- | --- | --- | --- | --- | --- | --- | --- | --- | --- |
|  |

| **Supplementary Table 2.** First site of recurrence | | | | | | |
| --- | --- | --- | --- | --- | --- | --- |
|  | Before PS matching | | | After PS matching | | |
|  | Open  (n=529) | LAP (n=1036) | *P* Value^1^ | Open (n=526) | LAP  (n=526) | *P* Value^1^ |
| First recurrent organ |  |  |  |  |  |  |
| Locoregional (anastomotic site） | |  | 0.761 |  |  | 0.506 |
| No | 526 (99.4%) | 1027 (99.1%) |  | 523 (99.4%) | 520 (98.9%) |  |
| Yes | 3 (0.6%) | 9 (0.9%) |  | 3 (0.6%) | 6 (1.1%) |  |
| Locoregional (Regional lymph nodes) | |  | 0.694 |  |  | >0.999 |
| No | 526 (99.4%) | 1032 (99.6%) |  | 523 (99.4%) | 524 (99.6%) |  |
| Yes | 3 (0.6%) | 4 (0.4%) |  | 3 (0.6%) | 2 (0.4%) |  |
| Locoregional (Other) |  |  | 0.235 |  |  | 0.124 |
| No | 525 (99.2%) | 1033 (99.7%) |  | 522 (99.2%) | 526 (100%) |  |
| Yes | 4 (0.8%) | 3 (0.3%) |  | 4 (0.8%) | 0 (0.0%) |  |
| Distant lymph node metastasis |  |  | 0.299 |  |  | 0.147 |
| No | 513 (97.0%) | 1014 (97.9%) |  | 510 (97.0%) | 518 (98.5%) |  |
| Yes | 16 (3.0%) | 22 (2.1%) |  | 16 (3.0%) | 8 (1.5%) |  |
| Liver |  |  | 0.914 |  |  | 0.808 |
| No | 494 (93.4%) | 969 (93.5%) |  | 491 (93.3%) | 488 (92.8%) |  |
| Yes | 35 (6.6%) | 67 (6.5%) |  | 35 (6.7%) | 38 (7.2%) |  |
| Lung |  |  | 0.320 |  |  | 0.681 |
| No | 499 (94.3%) | 990 (95.6%) |  | 496 (94.3%) | 500 (95.1%) |  |
| Yes | 30 (5.7%) | 46 (4.4%) |  | 30 (5.7%) | 26 (4.9%) |  |
| Hemostatic metastasis |  |  | >0.999 |  |  | >0.999 |
| No | 528 (99.8%) | 1034 (99.8%) |  | 525 (99.8%) | 525 (99.8%) |  |
| Yes | 1 (0.2%) | 2 (0.2%) |  | 1 (0.2%) | 1 (0.2%) |  |
| Peritoneal |  |  | 0.769 |  |  | 0.745 |
| No | 510 (96.4%) | 1002 (96.7%) |  | 508 (96.6%) | 505 (96.0%) |  |
| Yes | 19 (3.6%) | 34 (3.3%) |  | 18 (3.4%) | 21 (4.0%) |  |
| Bone |  |  | 0.552 |  |  | >0.999 |
| No | 529 (100.0%) | 1034 (99.8%) |  | 526 (100.0%) | 525 (99.8%) |  |
| Yes | 0 (0.0%) | 2 (0.2%) |  | 0 (0.0%) | 1 (0.2%) |  |
| Ovary |  |  | >0.999 |  |  | >0.999 |
| No | 529 (100.0%) | 1035 (99.9%) |  | 526 (100.0%) | 525 (99.8%) |  |
| Yes | 0 (0.0%) | 1 (0.1%) |  | 0 (0.0%) | 1 (0.2%) |  |
| Port site |  |  | >0.999 |  |  | >0.999 |
| No | 529 (100.0%) | 1035 (99.9%) |  | 526 (100.0%) | 525 (99.8%) |  |
| Yes | 0 (0.0%) | 1 (0.1%) |  | 0 (0.0%) | 1 (0.2%) |  |
| Other |  |  | >0.999 |  |  | >0.999 |
| No | 528 (99.8%) | 1034 (99.8%) |  | 525 (99.8%) | 524 (99.6%) |  |
| Yes | 1 (0.2%) | 2 (0.2%) |  | 1 (0.2%) | 2 (0.4%) |  |
| Unknown |  |  | >0.999 |  |  |  |
| No | 529 (100.0%) | 1035 (99.9%) |  | 526 (100.0%) | 526 (100.0%) |  |
| Yes | 0 (0.0%) | 1 (0.1%) |  | 0 (0.0%) | 0 (0.0%) |  |
| PS, propensity score.  ^1^Fisher’s Exact Test for Count Data | | | | | | |

Supplementary Table 3. Patient background by BMI stratification

|  | |  |  |  |  |  |
| --- | --- | --- | --- | --- | --- | --- |
|  | | |  |  |  |  |
| Characteristic | Open |  |  | LAP |  |  |
|  | BMI: 25-30 | BMI: ≥30 | d | BMI: 25-30 | BMI: ≥30 | d |
|  | n=462^a^ | n=64 |  | n=465^a^ | n=61 |  |
| Age, y, mean (SD) | 65.6 (9.4) | 62.3 (10.6) | 33.2 | 65.5 (9.1) | 63.4 (10.3) | 22.3 |
| Gender |  |  | 3.55 |  |  | 23.9 |
| Female | 194 (42.0%) | 28 (43.8%) |  | 189 (40.6%) | 32 (52.5%) |  |
| Male | 268 (58.0%) | 36 (56.3%) |  | 276 (59.4%) | 29 (47.5%) |  |
| BMI | 26.8 (1.3) | 32.6 (2.6) | 282 | 26.7 (1.3) | 32.7 (3.4) | 238 |
| Hypertension | |  | 0.528 |  |  | 17.8 |
| No | 237 (51.3%) | 33 (51.6%) |  | 247 (53.1%) | 27 (44.3%) |  |
| Yes | 225 (48.7%) | 31 (48.4%) |  | 218 (46.9%) | 34 (55.7%) |  |
| Diabetes mellitus | |  | 0.751 |  |  | 11.4 |
| No | 348 (75.3%) | 48 (75.0%) |  | 344 (74.0%) | 42 (68.9%) |  |
| Yes | 114 (24.7%) | 16 (25.0%) |  | 121 (26.0%) | 19 (31.1%) |  |
| Cerebrovascular disease | |  | 22.2 |  |  | 19.4 |
| No | 424 (91.8%) | 62 (96.9%) |  | 437 (94.0%) | 54 (88.5%) |  |
| Yes | 38 (8.2%) | 2 (3.1%) |  | 28 (6.0%) | 7 (11.5%) |  |
| Respiratory disease | |  | 10.8 |  |  | 11.1 |
| No | 426 (92.2%) | 57 (89.1%) |  | 427 (91.8%) | 54 (88.5%) |  |
| Yes | 36 (7.8%) | 7 (10.9%) |  | 38 (8.2%) | 7 (11.5%) |  |
| Cardiovascular disease | |  | 7.54 |  |  | 4.37 |
| No | 402 (87.0%) | 54 (84.4%) |  | 405 (87.1%) | 54 (88.5%) |  |
| Yes | 60 (13.0%) | 10 (15.6%) |  | 60 (12.9%) | 7 (11.5%) |  |
| pT |  |  | 2.34 |  |  | 1.11 |
| 0 | 24 (5.2%) | 3 (4.7%) |  | 24 (5.2%) | 3 (4.9%) |  |
| 1 | 438 (94.8%) | 61 (95.3%) |  | 441 (94.8%) | 58 (95.1%) |  |
| pN |  |  | 10.8 |  |  | 5.3 |
| 0 | 235 (50.9%) | 36 (56.3%) |  | 241 (51.8%) | 30 (49.2%) |  |
| 1 | 227 (49.1%) | 28 (43.8%) |  | 224 (48.2%) | 31 (50.8%) |  |
| Sideness |  |  | 1.71 |  |  | 1.22 |
| Right | 191 (41.3%) | 27 (42.2%) |  | 201 (43.2%) | 26 (42.6%) |  |
| Left | 271 (58.7%) | 37 (57.8%) |  | 264 (56.8%) | 35 (57.4%) |  |
| Conversion to open surgery |  |  |  |  |  |  |
| No | NA | NA |  | 451 (97.0%) | 53 (86.9%) | 37.8 |
| Yes | NA | NA |  | 14 (3.0%) | 8 (13.1%) |  |

d, absolute standardized difference; SD, standard deviation; NA, not applicable.

Supplementary Table 4. Postoperative outcomes by BMI stratification

|  | |  |  |  |  |  |  |  |
| --- | --- | --- | --- | --- | --- | --- | --- | --- |
|  | | |  |  |  |  |  |  |
|  | Characteristic | Open |  |  |  | LAP |  |  |
|  |  | BMI: 25-30 | BMI: ≥30 | *P* value^1^ |  | BMI: 25-30 | BMI: ≥30 | *P* value^1^ |
|  |  | n=462 | n=64 |  |  | n=465^1^ | n=61 |  |
|  | Overall(G3) |  |  | 0.077 |  |  |  | 0.751 |
|  | No | 439 (95.0%) | 57 (89.1%) |  |  | 444 (95.5%) | 58 (95.1%) |  |
|  | Yes | 23 (5.0%) | 7 (10.9%) |  |  | 21 (4.5%) | 3 (4.9%) |  |
|  | Anastomotic leakage (G3) |  |  | 0.319 |  |  |  | >0.999 |
|  | No | 460 (99.6%) | 62 (96.8%) |  |  | 456 (98.1%) | 60 (98.4%) |  |
|  | Yes | 2 (0.4%) | 1 (1.6%) |  |  | 9 (1.9%) | 1 (1.6%) |  |
|  | Unknown | 0 | 1 (1.6%) |  |  |  |  |  |
|  | Ileus (Overall, G3) |  |  | >0.999 |  |  |  | >0.999 |
|  | No | 452 (97.8%) | 63 (98.4%) |  |  | 461 (99.1%) | 61 (100.0%) |  |
|  | Yes | 10 (2.2%) | 1 (1.6%) |  |  | 4 (0.9%) | 0 (0.0%) |  |
|  | Obstructive ileus (G3) |  |  | 0.406 |  |  |  | >0.999 |
|  | No | 459 (99.4%) | 63 (98.4%) |  |  | 462 (99.4%) | 61 (100.0%) |  |
|  | Yes | 3 (0.6%) | 1 (1.6%) |  |  | 3 (0.6%) | 0 (0.0%) |  |
|  | Paralytic ileus (G3) |  |  | >0.999 |  |  |  | >0.999 |
|  | No | 455 (98.5%) | 64 (100.0%) |  |  | 464 (99.8%) | 61 (100.0%) |  |
|  | Yes | 7 (1.5%) | 0 (0.0%) |  |  | 1 (0.2%) | 0 (0.0%) |  |
|  | 30-day mortality |  |  |  |  |  |  |  |
|  | No | 461 (99.8%) | 64 (100.0%) |  |  | 465 (100.0%) | 61 (100.0%) |  |
|  | Unknown | 1 (0.2%) | 0 |  |  |  |  |  |
|  | Length of hospital stay(day) |  |  | 0.935 |  |  |  | 0.602 |
|  | Median | 12.0 | 13.0 |  |  | 10.0 | 10.0 |  |
|  | (IQR) | (10.0, 16.0) | (10.0, 15.0) |  |  | (8.0, 14.0) | (8.0, 13.0) |  |
|  | (Range) | (5.0, 384.0) | (6.0, 68.0) |  |  | (0, 575.0) | (6.0, 37.0) |  |
|  | Postop　chemotherapy |  |  | 0.790 |  |  |  | 0.892 |
|  | No | 226 (48.9%) | 33 (51.6%) |  |  | 236 (50.8%) | 30 (49.2%) |  |
|  | Yes | 236 (51.1%) | 31 (48.4%) |  |  | 229 (49.2%) | 31 (50.8%) |  |

^1^Fisher’s Exact Test; Wilcoxon rank sum test

**Supplementary Figure 1A.** Kaplan-Meier curve of relapse- free survival divided into 4 groups by surgical approach and BMI (25-30, ≥30).


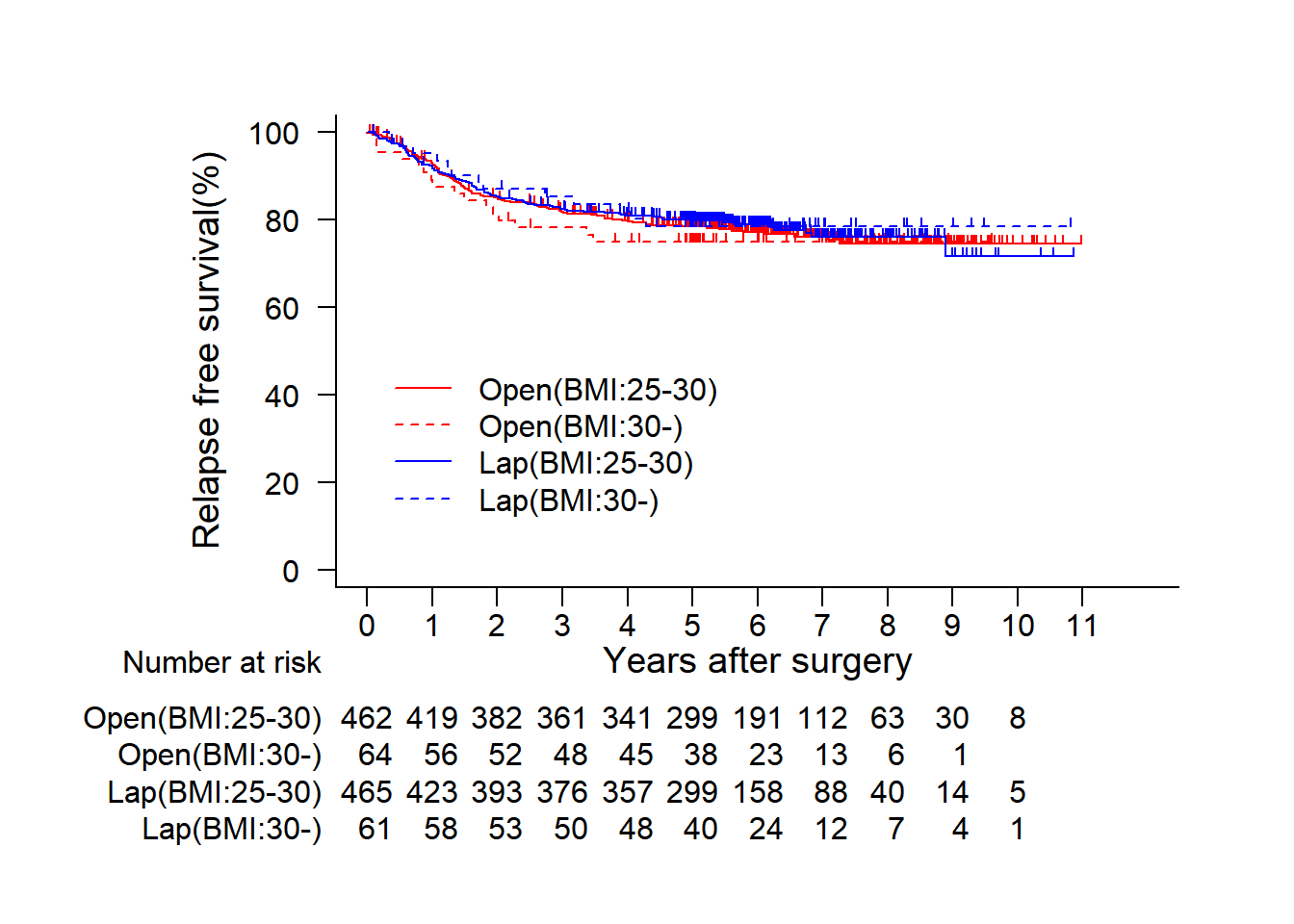


*P=0.898*

Relapse- free survival (%)

Open, open surgery; LAP, laparoscopic surgery; C, cecum; A, ascending colon; T, transverse colon; D, descending colon; S, sigmoid colon; RS, rectosigmoid colon

**Supplementary Figure 1B.** Kaplan-Meier curve of overall survival divided into 4 groups by surgical approach and BMI (25-30, ≥30).


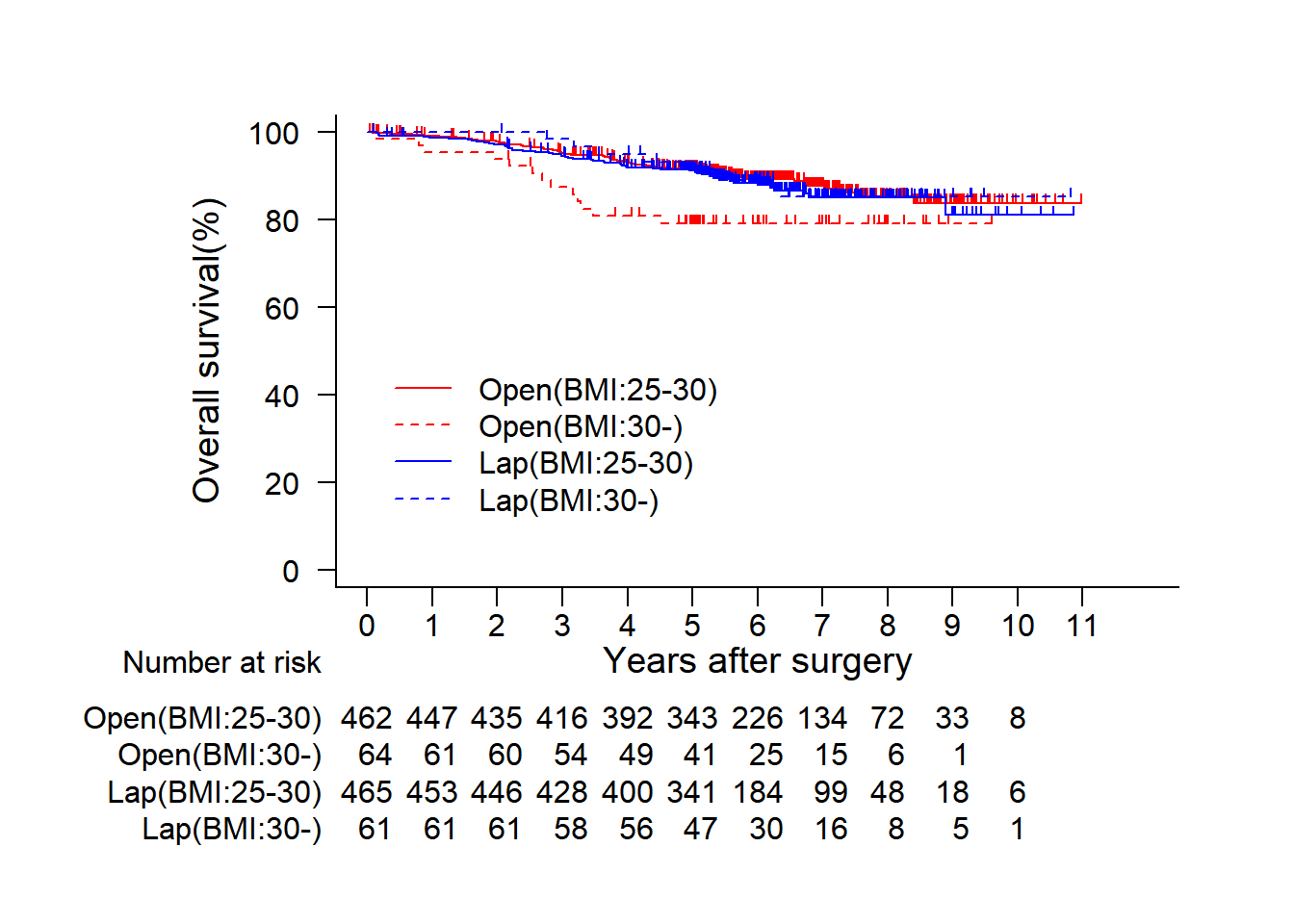


*P=0.128*

Overall survival (%)

Open, open surgery; LAP, laparoscopic surgery; C, cecum; A, ascending colon; T, transverse colon; D, descending colon; S, sigmoid colon; RS, rectosigmoid colon

Supplementary Figure 2A. Kaplan-Meier curve of relapse- free survival divided into 4 groups by surgical approach and tumor location.


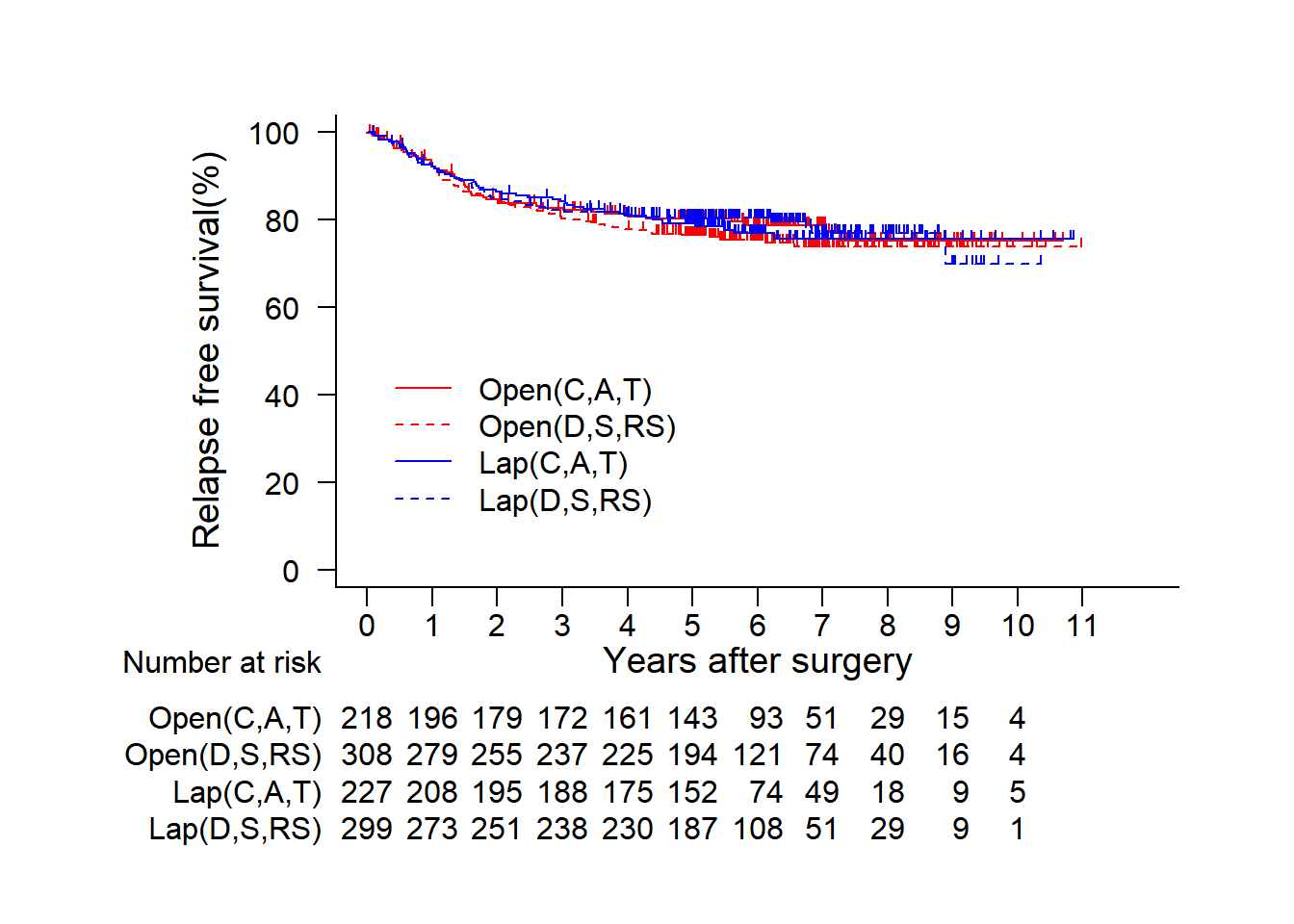


Relapse- free survival (%)

*P=0.781*

Open, open surgery; LAP, laparoscopic surgery; C, cecum; A, ascending colon; T, transverse colon; D, descending colon; S, sigmoid colon; RS, rectosigmoid colon

**Supplementary Figure 2B.**

Kaplan-Meier curve of overall survival divided into 4 groups by surgical approach and tumor location.


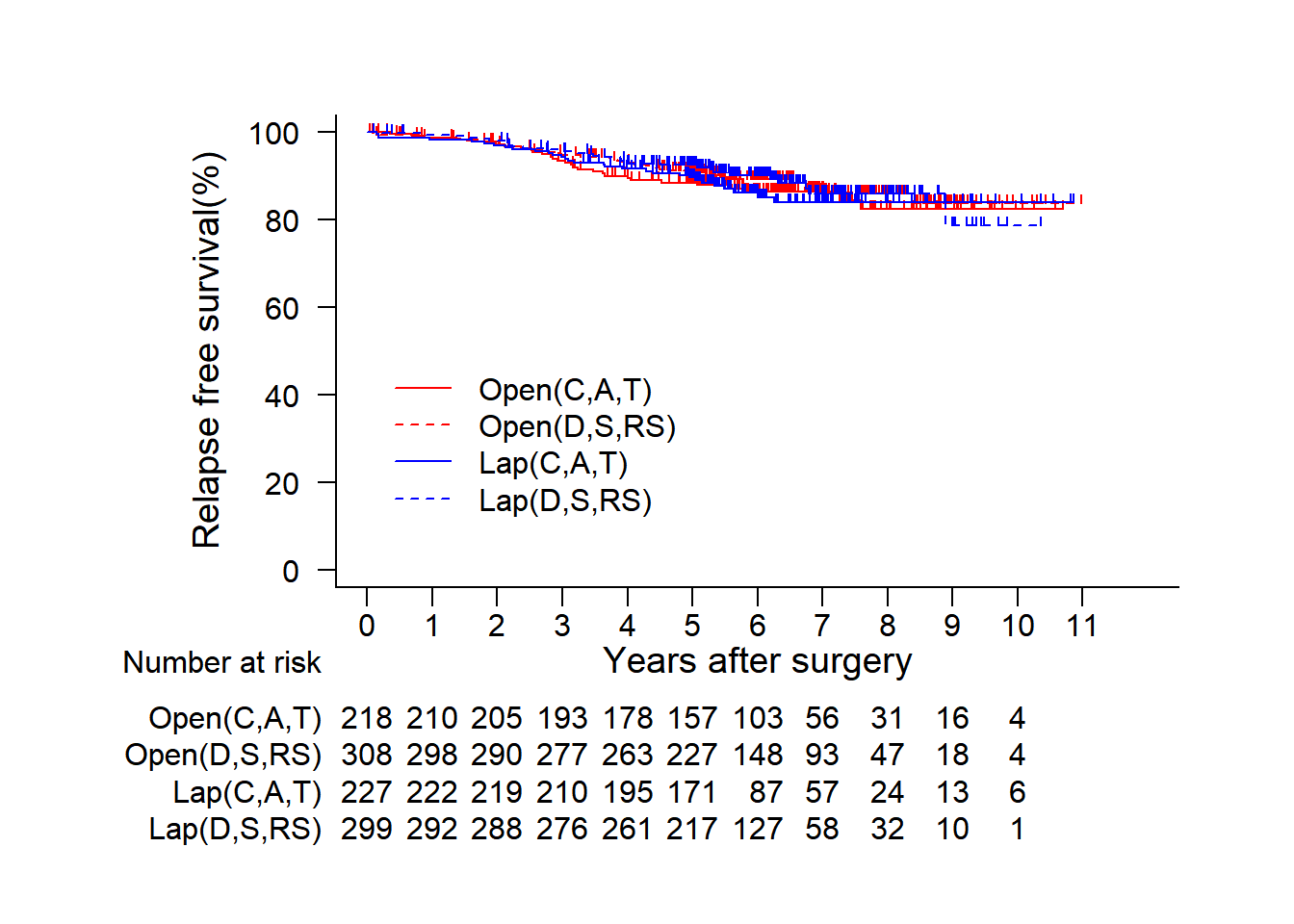


Overall survival (%)

*P=0.711*

Open, open surgery; LAP, laparoscopic surgery; C, cecum; A, ascending colon; T, transverse colon; D, descending colon; S, sigmoid colon; RS, rectosigmoid colon
